# Cost-efficiency of specialist inpatient rehabilitation for working-aged adults with complex neurological disabilities: a multicentre cohort analysis of a national clinical data set

**DOI:** 10.1136/bmjopen-2015-010238

**Published:** 2016-02-24

**Authors:** Lynne Turner-Stokes, Heather Williams, Alan Bill, Paul Bassett, Keith Sephton

**Affiliations:** 1 Faculty of Life Sciences and Medicine, Department of Palliative Care, Policy and Rehabilitation, King's College London, London, UK; 2 Regional/Hyper-acute Rehabilitation Unit, Northwick Park Hospital, UK; 3 Statsconsultancy Ltd, London, UK

**Keywords:** outcome measurement, dependency, cost-efficiency

## Abstract

**Objectives:**

To evaluate functional outcomes, care needs and cost-efficiency of specialist rehabilitation for a multicentre cohort of inpatients with complex neurological disability, comparing different diagnostic groups across 3 levels of dependency.

**Design:**

A multicentre cohort analysis of prospectively collected clinical data from the UK Rehabilitation Outcomes Collaborative (UKROC) national clinical database, 2010–2015.

**Setting:**

All 62 specialist (levels 1 and 2) rehabilitation services in England.

**Participants:**

Working-aged adults (16–65 years) with complex neurological disability. Inclusion criteria: all episodes with length of stay (LOS) 8–400 days and complete outcome measures recorded on admission and discharge. Total N=5739: acquired brain injury n=4182 (73%); spinal cord injury n=506 (9%); peripheral neurological conditions n=282 (5%); progressive conditions n=769 (13%).

**Intervention:**

Specialist inpatient multidisciplinary rehabilitation.

**Outcome measures:**

Dependency and care costs: Northwick Park Dependency Scale/Care Needs Assessment (NPDS/NPCNA). Functional independence: UK Functional Assessment Measure (UK Functional Independence Measure (FIM)+FAM). Cost-efficiency: (1) time taken to offset rehabilitation costs by savings in NPCNA-estimated costs of ongoing care, (2) FIM efficiency (FIM gain/LOS days), (3) FIM+FAM efficiency (FIM+FAM gain/LOS days). Patients were analysed in 3 groups of dependency.

**Results:**

Mean LOS 90.1 (SD 66) days. All groups showed significant reduction in dependency between admission and discharge on all measures (paired t tests: p<0.001). Mean reduction in ‘weekly care costs’ was greatest in the high-dependency group at £760/week (95% CI 726 to 794)), compared with the medium-dependency (£408/week (95% CI 370 to 445)), and low-dependency (£130/week (95% CI 82 to 178)), groups. Despite longer LOS, time taken to offset the cost of rehabilitation was 14.2 (95% CI 9.9 to 18.8) months in the high-dependency group, compared with 22.3 (95% CI 16.9 to 29.2) months (medium dependency), and 27.7 (95% CI 15.9 to 39.7) months (low dependency). FIM efficiency appeared greatest in medium-dependency patients (0.54), compared with the low-dependency (0.37) and high-dependency (0.38) groups. Broadly similar patterns were seen across all 4 diagnostic groups.

**Conclusions:**

Specialist rehabilitation can be highly cost-efficient for all neurological conditions, producing substantial savings in ongoing care costs, especially in high-dependency patients.

**Strengths and limitations of this study**

- A large 5-year national consecutive cohort analysis representing all specialist (levels 1 and 2) rehabilitation units in England.
- Prospective routinely collected data are reflective of real clinical practice.
- Different methods for evaluation of cost-efficiency are compared in the same data set.
- Owing to evolution of reporting requirements over the data collection period, the outcomes of interest were collected in less than 50% of the full rehabilitation data set, so selection bias cannot be excluded.
- This highly selected group of patients with complex needs is atypical in comparison to populations described in published analyses from other large data sets, but has potential relevance for other health systems that provide tertiary specialist rehabilitation services.

## Introduction

Over 1 million people in the UK (2% of the population) have a disabling neurological condition, of which 350 000 require help for most of their daily activities and it is estimated that 850 000 people care for someone with a neurological condition.^1^ By improving independence and autonomy, rehabilitation has the potential to reduce the needs for care and thus relieve the burden and costs of care, both for family and society. Although there is a growing body of trial-based evidence for the effectiveness of rehabilitation in a variety of neurological conditions,^2^ ^3^ there are other important questions that require a practice-based approach to determine what works best for which patients and what approaches represent value for money in the context of real-life clinical practice.^4^ ^5^

Much of the evidence for effectiveness of rehabilitation comes from the arenas of stroke and care of older people. To date, there has been relatively little focus on younger (ie, working aged) adults with complex disability following neurological illness or injury. Specialist rehabilitation is increasingly recognised as an essential component of healthcare for this group of patients.^6^ However, it can be a costly intervention and systematic evaluation is required to demonstrate that programmes are both effective and cost-efficient. Porter and Teisberg^7^ introduced the concept of ‘value-based healthcare’, where the goal is not necessarily to minimise costs but to maximise ‘value’, defined as ‘patient outcomes divided by costs’.

The Functional Independence Measure (FIM) is the most widely used standardised outcome measure for rehabilitation in the world. Established large rehabilitation data sets in the USA and Australia rely on the FIM, not only as a measure of functional gains during rehabilitation, but as a casemix tool and a measure of cost-efficiency. In the absence of direct costing data, the ‘FIM-efficiency index’ (FIM gain/length of stay (LOS)) is often used as a proxy for cost-efficiency.^8–13^ However, such estimations have a number of weaknesses:

1. They assume linearity of change and equal weighting of items to the prediction of overall cost of care, which is not necessarily the case.
2. They are frequently confounded by floor and ceiling effects.^14^
3. The FIM is largely focused on physical disability, which limits its use in the context of complex neurological disability, where cognitive and psychosocial problems are often the principal limiting factors.

The UK National Health Service (NHS) provides one of the most comprehensive health and social service systems in the world^15^ and demands a somewhat different approach.

- Rehabilitation services are planned and provided in coordinated regional networks over a relatively small geographical area. Local general (level 3) rehabilitation services provide for the majority of patients, but a smaller number are referred to specialist (level 1 or 2) services, which take a selected population of mainly younger adults with complex needs for rehabilitation that are beyond the scope of their local rehabilitation services.^16^
- The statutory commitment to life-long provision of care supports longer periods of rehabilitation in these specialist services, provided that this can be demonstrated to produce meaningful cost-benefits through gains in wider independence and reduction of long-term care needs.

Since 2010, the national UK Rehabilitation Outcomes Collaborative (UKROC) database has collated episode data for all inpatients admitted to specialist rehabilitation services (levels 1 and 2) in England, providing national benchmarking on quality, outcomes and cost-efficiency of rehabilitation. Within the UKROC data set, functional gain is evaluated using the UK Functional Assessment Measure (UK FIM+FAM),^17^ ^18^ which extends the FIM to provide greater coverage of cognitive and psychosocial function. Cost-efficiency is computed in terms of the length of time taken to offset the initial costs of rehabilitation through savings in the ongoing costs of community care as estimated by the Northwick Park Dependency Care Needs Assessment.^19^ ^20^

A previously published single-centre analysis using these indices demonstrated the cost-efficiency of rehabilitation for younger adults with complex needs following acquired brain injury (ABI),^21^ and showed that longer lengths of stay can provide value for money by reducing ongoing care costs.^22^ The cost-benefits were particularly marked for highly dependent patients, while ‘FIM efficiency’ appeared to be greatest for the medium-dependency group. This finding was important as highly dependent patients may be denied rehabilitation in other healthcare systems on the basis that they are costly to care for and not expected to make significant gains on the FIM.^21^

The objective of this article is to present the first national cohort analysis of the UKROC database to describe functional outcome, change in care needs and cost-efficiency following specialist rehabilitation for working-aged adults with complex disability arising from neurological conditions. In particular, we wished to determine whether the single-centre findings above were reproducible across multiple centres and across a wider range of neurological conditions.

Specific research questions were:

1. What types of functional gain are made during rehabilitation by patients with different neurological conditions?
2. Can longer lengths of stay for highly dependent patients be justified by savings in ongoing care costs?
3. Are there important differences in outcome and cost-efficiency across different neurological conditions and for different levels of patient dependency that service planners should be aware of?

## Methods

### Design

A large 5-year multicentre national cohort analysis of prospectively collected clinical data from the UKROC national clinical database 2010–2015. Participants were working-aged adults (aged 16–65) with complex neurological disability undergoing specialist inpatient rehabilitation in England.

### Setting and data source

In England, level 1 rehabilitation units are tertiary services providing for a regionally based catchment population of 3–5million and taking a highly selected caseload of patients with very complex needs. They are subdivided by casemix into hyperacute, 1a (physical disability), 1c (cognitive behavioural) and 1b (mixed) services. Level 2 services take a mixed caseload providing for a more local population, divided into 2a (supradistrict) and 2b (local district) specialist rehabilitation services. The data reporting requirements have evolved over time and vary somewhat between the different levels of service.

The UKROC database was established in 2009 through funding a programme grant from the UK National Institute for Health Research (NIHR),^23^ but now provides the national commissioning data set for NHS England. The database collates de-identified data, which are uploaded at monthly intervals and stored on a secured NHS server held at Northwick Park Hospital. It is overseen by a steering group of the British Society of Rehabilitation Medicine.

The data set comprises sociodemographic and process data (waiting times, discharge destination, etc) as well as clinical information on rehabilitation needs, inputs and outcomes. Full details may be found on the UKROC website http://www.csi.kcl.ac.uk/ukroc.html.

- Data collection started formally in April 2010. Reporting was initially voluntary and contributing centres could report any one of three approved outcome measures, the Barthel Index (BI), the FIM or UK FIM+FAM.
- Since April 2012, levels 1 and 2a services are commissioned centrally by NHS England and are required to report the full UKROC data set for all admitted episodes, including the UK FIM+FAM as the principal outcome measure.
- Reporting of the Northwick Park Dependency Scale and Care Needs Assessment as a measure of cost-efficiency was optional until April 2013, but is now a requirement for national benchmarking for these levels 1 and 2a services.
- Locally commissioned level 2b (local district) services may still report only lower level data such as the BI or FIM.

### Measurements

*The UK FIM+FAM* is a global measure of disability.^17^ ^18^ It includes the 18-item FIM (V.4) and adds a further 12 items, mainly addressing psychosocial function giving a total of 30 items (16 motor and 14 cognitive items). Each item is scored on a seven-point ordinal scale from 1 (total dependence) to 7 (complete independence). Further details are published elsewhere.^17^ ^18^

*The Northwick Park Dependency Score (NPDS)* is an ordinal scale of dependency on nursing staff time (number of helpers and time taken to assist with each task) designed to assess needs for care and nursing in clinical rehabilitation settings.^19^ It comprises a 16-item scale of basic care needs (range 0–65) and a 7-item scale of special nursing needs (range 0–35)—total range 0–100. It is shown to be a valid and reliable measure of needs for care and nursing in rehabilitation settings.^24^ It supports categorisation of patients into three dependency groups based on their admission NPDS:^21^

- Low dependency (NPDS <10): patients are largely independent for basic self-care,
- Medium (NPDS 10–24): patients generally require help from one person for most self-care tasks,
- High (NPDS ≥25): patients require help from two or more persons for most care tasks and often also have special nursing needs.

The NPDS also translates via a computerised algorithm to the Northwick Park Care Needs Assessment (NPCNA)^20^ which estimates the total care hours per week and the approximate weekly cost of care (£/week) in the community, based on the UK care agency costs. The NPCNA provides a generic assessment of care needs, regardless of who provides and pays for them. The estimated cost of care is therefore independent of individual circumstances or local policy for the provision continuing care, which varies widely across the UK. The algorithm is embedded within the UKROC software and generates this information automatically.

Although there is no formal accreditation process for use of the UK FIM+FAM and NPDS, the attendance of UK FIM+FAM training by at least a core team of staff is requirement for UKROC registration. All units that are registered with UKROC have access to the national training and update workshops, as well as free telephone support.

### Cost-efficiency of rehabilitation

Within the UKROC data set, the cost-efficiency is calculated as the time taken to offset the cost of rehabilitation by the resulting savings in the cost of ongoing care in the community. This is calculated from the ‘episode cost of rehabilitation’ divided by ‘reduction in weekly cost of care’ from admission to discharge, as estimated by the NPCNA. The episode cost was calculated per patient as ‘bed-day cost×LOS’. The cost per bed-day was calculated on updated data from our previously published cost-analysis.^25^ We used mean per diem costs for the different levels of service as follows: 1 hyperacute: £670, 1a: £540, 1b: £483, 1c: £634, 2a: 452, 2b: £418. For comparison with other series, we also report FIM efficiency, calculated at individual patient level as change in total FIM score/LOS in days. FIM+FAM efficiency is calculated as change in total UK FIM+FAM score/LOS in days.

### Valid LOS

In order to identify plausible admissions for rehabilitation (as opposed to brief inpatient assessment or for long-term care) we selected patients with LOS between 8 and 400 days. Other cohort studies have used similar cut-off points,^26^ although the exact time frames may vary according with local practice. In this cohort, we excluded patients staying for 1 week or less as they would not meet even the lowest time-thresholds for repeat assessment. The NHS England service specification for rehabilitation stipulates a maximum programme length of 180 days with a trim point of 14 days (ie, 194 days in total). Subject to approval, extension for a second period may be granted in some cases if it can be justified on the grounds of anticipated functional gain and cost-efficiency, bringing the total allowed LOS to 388 days. Allowing for possible short delays in discharge at the end of programme, we therefore set 400 days as the ceiling for a plausible LOS for rehabilitation.

### Data extraction

De-identified data were extracted for all recorded inpatient episodes for adults aged 16–65 years admitted to level 1 or 2 specialist rehabilitation service and discharged during the 5-year period between 1.4.2010 and 31.3.15, if they had:

- A neurological condition recorded in the diagnostic category;
- A LOS 8–400 days;
- Valid UK FIM+FAM and NPDS ratings completed both within 10 days of admission and within the last week before discharge.

Data were collated in MS Excel and transferred to SPSS V.22 for analysis.

### Data handling and analysis

Because data reporting was initially voluntary, missing data were expected. No data were imputed for missing values. There is continued debate about whether to use parametric or non-parametric statistics for this type of data. In this analysis, given the large size of the data set and long ordinal nature of the measures (ie, many possible data points), we have elected to describe and analyse the data using parametric statistics—although non-parametric analysis gave very similar results and is available from the authors if required.

- Ninety-five per cent CIs were calculated and multiple comparisons made using bootstrapping with samples of n=1000, to minimise the effect of any skewed data.
- Paired t tests were used to compare significant differences between admission and discharge.
- One-way analyses of variances (ANOVAs) with bootstrapped post hoc analysis and Bonferroni correction to correct for multiple tests were used to compare differences for diagnostic groups and for different levels of dependency. Key results from post hoc analyses are summarised in the text, but not given in tables. Further details are available on request from the corresponding author.

In this non-interventional observational study, size was not predetermined but dictated by the accruals to the national data set over the 5-year period that met the inclusion criteria. Because the data set was dominated by patients with ABI, analysis was also undertaken separately for each diagnostic group.

## Results

**Figure 1:**
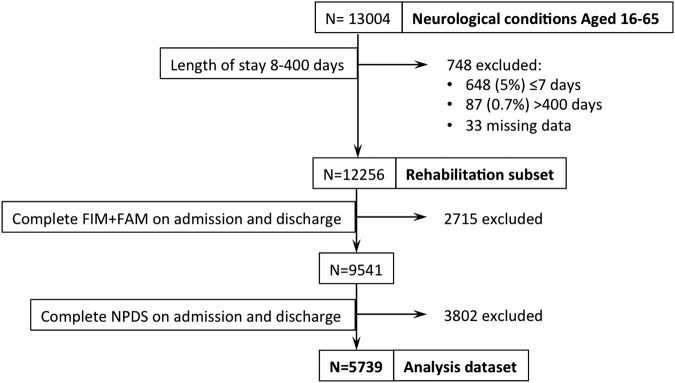
The data extraction process to derive the data set used for analysis. FIM, Functional Independence Measure; FIM+FAM, UK Functional Assessment Measure; NPDS, Northwick Park Dependency Score.

Figure 1 illustrates the data extraction process. From a total of 13 004 registered episodes for adults aged 16–65 with a neurological condition, 12 256 had a LOS between 8 and 400 days representing the data set of adults admitted for rehabilitation. Of these, 5739 (47%) had a valid NPDS and FIM+FAM on both admission and discharge and were included in the analysed sample.

A total of 62 rehabilitation units (15 level 1, 15 level 2a and 32 level 2b services) provided data, with good representation across all four health regions in England.

Demographics are given in table 1. Because the sample comprised less than 50% of the total rehabilitation data set, demographics were compared for the analysed and the total sample. No significant differences were found.

**Table 1:** Demographics of the total analysed population and for the four main diagnostic groups

Parameter	Missing n=	All N=5739	ABI N=4182 (73%)	SCI N=506 (9%)	Peripheral N=282 (5%)	Progressive N=769 (13%)	Full data set N=12 256 *
Age							
Mean (SD)	0	47.3 (12.6)	46.8 (12.8)	49.3 (12.7)	47.8 (12.8)	48.6 (10.8)	47.0 (12.8)
M:F ratio (%)	4	59/41%	62/38%	59/41%	55/45%	40/60%	60/40%
Time since onset (days)							
Mean (SD)		657 (2093)	237 (1196)	660 (2763)	139 (359)	3223 (3576)	691 (2273)
Median (IQR)		59 (29–137)	54 (28–104)	48 (25–136)	60 (30–11)	2326 (90–5031)	57 (28–133)
Length of stay (days)							
Mean (SD) days	0	90.1 (65.5)	90.7 (67.4)	72.8 (58.5)	79.9 (60.6)	56.3 (60.0)	79.2 (67.3)
Cost of episode							
Mean (SD)	0	£39 381 (£32 235)	£43 053 (£33 473)	£32 813 (£26 519)	£36 631 (£31.357)	£24 739 (£22 857)	£37 158 (£33 121)
Diagnostic subcategories n (%)							
Trauma		1259 (21.9)	1127 (26.9)	125 (24.7)	7 (2.5)		2769 (22.6)
Vascular		2048 (35.7)	1979 (47.7)	49 (9.7)	20 (7.1)		4299 (35.1)
Inflammatory		448 (7.8)	175 (4.2)	109 (21.5)	164 (58.2)		950 (7.7)
Tumour		347 (6.0)	268 (6.4)	79 (15.6)	–		705 (5.8)
Other		934 (16.3)	595 (14.3)	140 (27.7)	89 (31.6)	110 (14.3)	1864 (15.3)
Multiple sclerosis		636 (11.1)				636 (82.7)	1323 (10.8)
Motor neurone disease		7 (0.1)				7 (0.9)	16 (0.1)
Parkinson's disease		13 (0.2)				13 (1.7)	23 (0.2)
Missing		47 (0.8)	38 (0.9)	4 (0.8)	2 (0.7)		307 (2.5)

No significant differences were seen between the demographics of the analysis dataset and the full data set.

*N=12 256 is made up of 9000 (73%) ABI, 977 (8%) SCI, 642 (5%) peripheral and 1637 (13%) progressive conditions.

ABI, acquired brain injury; F, female; M, male; SCI, spinal cord injury.

The study sample comprised approximately 3:2 males:females, with a mean age at admission of 47.3 (SD=12.6) years. The mean rehabilitation LOS was 90.1 (SD=65.5) days. Nearly three-quarters of the sample (73%) had ABI, the remainder having spinal cord injuries (SCIs; 9%), peripheral neurological conditions, for example, Guillain-Barré syndrome (5%) and progressive conditions (13%). Table 1 shows the demographics for these diagnostic groups and shows the breakdown of aetiological causes within each category. As the time between onset and admission (‘time since onset’) was very highly skewed, the median and IQR is given as well as the mean (SD). Excluding the progressive conditions, the mean time since onset for ABI, SCI and peripheral neurological conditions was 9.0 months (SD 46.5).

One-way ANOVA tests confirmed significant differences in LOS and episode costs (p<0.001) between the different diagnostic groups. Patients with ABI stayed longest (mean 90 days) with the highest episode costs (mean approximately £43 000), while those with progressive conditions stayed the shortest (mean 56 days) and corresponding lower episode costs (mean approximately £25 000).

### Dependency and functional outcomes

Table 2 summarises the overall dependency and functional outcome scores for the sample, together with cost-efficiency. Between admission and discharge there was highly significant increase in all parameters of functional independence (FIM+FAM; p<0.001), with corresponding reduction in all parameters of dependency (NPDS/NPCNA; p<0.001). The mean total FIM+FAM gain was 35.5 and the mean individually calculated FIM+FAM efficiency/week was 0.67 (95% CI 0.64 to 0.69). The mean total cost of the rehabilitation programme was £39 381 and mean savings in ongoing cost of care in the community was £496/week. The mean time taken to offset the initial costs of rehabilitation was 17.9 months (95% CI 14.5 to 21.4).

**Table 2:** Overall dependency and functional outcome scores on admission and discharge (n=5739)

	Admission Mean (SD)	Discharge Mean (SD)	Mean difference	95% CIs*	t	p Value Two-tailed
Functional independence (FIM+FAM)						
Self-care	26.2 (13.0)	34.7 (13.4)	8.6	8.3 to 8.8	71.6	<0.001
Sphincter	7.2 (4.8)	9.7 (4.8)	2.5	2.4 to 2.6	50.3	<0.001
Transfers	10.8 (8.1)	17.7 (9.2)	7.0	6.7 to 7.1	72.6	<0.001
Locomotion	6.4 (4.7)	10.9 (6.0)	4.6	4.5 to 4.7	71.1	<0.001
Communication	21.9 (10.2)	26.1 (9.2)	4.2	4.1 to 4.4	54.4	<0.001
Psychosocial	16.2 (7.4)	19.9 (6.9)	3.7	3.5 to 4.8	54.4	<0.001
Cognition	19.8 (10.4)	24.7 (9.6)	5.0	4.8 to 5.1	57.6	<0.001
Subscale and total scores FIM+FAM						
Motor	50.6 (27.9)	72.9 (31.6)	22.7	22.1 to 23.3	79.7	<0.001
Cognitive	58.0 (26.0)	70.8 (24.2)	12.8	12.5 to 13.3	64.6	<0.001
Total FIM+FAM	108.5 (47.1)	143.7 (51.0)	35.5	34.6 to 36.4	83.8	<0.001
Subscale and total scores FIM only†						
Motor	41.5 (24.2)	59.9 (26.7)	18.4	17.9 to 18.8	76.7	<0.001
Cognitive	21.7 (10.0)	25.9 (9.0)	4.2	4.0 to 4.3	56.5	<0.001
Total FIM	63.1 (30.2)	85.8 (33.1)	22.6	22.1 to 23.1	80.5	<0.001
Dependency (NPDS/NPCNA)						
Total NPDS score	31.0 (17.4)	20.8 (17.6)	−10.3	−10.7 to −10.0	−59.6	<0.001
Care hours/week	44.7 (19.5)	31.7 (21.2)	−13.0	−13.4 to −12.6	−59.2	<0.001
Care costs/week	£1580 (£933)	£1083 (£950)	−£496	−£517 to −£475	−45.9	<0.001
Cost-efficiency parameters						
			Mean	95% CI		
FIM efficiency			0.42	0.41 to 0.44		
FIM+FAM efficiency			0.67	0.64 to 0.69		
Time to offset the costs of rehabilitation (months)			17.9	14.5 to 21.4		

*Bootstrapped CIs based on 1000 bootstrap samples.

†FIM sores are provided for comparison with other series.

FIM, Functional Independence Measure; FIM+FAM, UK Functional Assessment Measure; NPDS, Northwick Park Dependency Score; NPCNA, Northwick Park Care Needs Assessment.

### Differences between diagnostic groups

**Figure 2:**
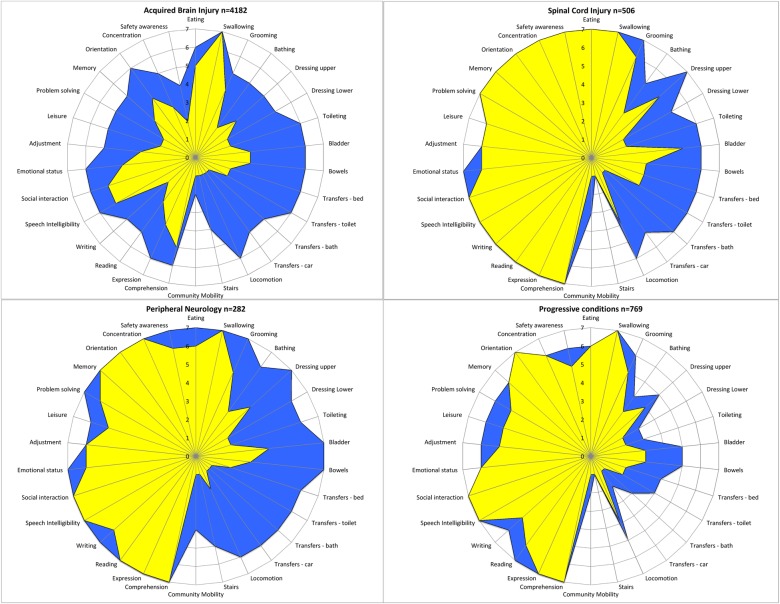
The radar chart (or ‘FAM splat’) provides a graphic representation of the disability profile from the FIM+FAM data. The 30-scale items are arranged as spokes of a wheel. Scoring levels from 1 (total dependence) to 7 (total independence) run from the centre outwards. Thus, a perfect score would be demonstrated as a large circle. This composite radar chart illustrates the median scores on admission and discharge. The yellow-shaded portion represents the median scores on admission for each item. The blue-shaded area represents the change in median score from admission to discharge. Clear differences in the pattern of disability can be seen between the four groups. FIM, Functional Independence Measure; FIM+FAM, UK Functional Assessment Measure.

The UKROC software generates ‘FAM splats’ in the form of radar charts which provide an ‘at a glance’ view of the disability profile and patterns of change during rehabilitation for the 30 FIM+FAM items. Figure 2 shows the composite FAM splats based on median item scores at admission and discharge for the four main diagnostic groups. They illustrate the clinical value of recording change in psychosocial, as well as physical function, which would not be detected by changes in the FIM items alone.

The differences in functional outcome across the diagnostic groups are summarised in table 3. On admission, FIM+FAM motor scores were broadly similar across all the categories with the difference only crossing the threshold for significance between the ABI and SCI groups. However, as may be expected, cognitive FIM+FAM scores were significantly lower in ABI than all other diagnosis (p<0.001), and remained so at discharge despite the substantially greater change in this group (mean 15.7). Cognitive FIM+FAM scores were also significantly lower for progressive conditions than for the SCI and peripheral neurology groups, but the latter were similar.

**Table 3:** Comparison of functional and dependency scores between diagnostic groups

Parameter	ABI (n=4182)		SCI (n=506)		Peripheral (n=282)		Progressive (n=769)		One-way ANOVA*	
	Mean	95% CI	Mean	95% CI	Mean	95% CI	Mean	95% CI	F	p Value
*UK FIM+FAM*									*Between groups*	
Admission										
Motor	50.1	49.2 to 51.0	57.1	55.2 to 58.9	51.2	48.6 to 54.0	52.8	51.0 to 54.4	11.2	<0.001
Cognitive	50.7	50.0 to 54.1	86.2	85.1 to 87.3	81.8	79.9 to 83.6	74.2	72.7 to 75.6	582.5	<0.001
Total	100.8	99.3 to 102.3	143.3	140.7 to 145.8	133.0	129.2 to 136.9	127.0	124.2 to 129.6	201.5	<0.001
Discharge										
Motor	74.0	73.0 to 74.9	81.2	79.3 to 83.3	85.0	82.0 to 87.8	64.3	62.3 to 66.2	49.5	<0.001
Cognitive	66.4	65.6 to 67.1	90.7	88.9 to 91.5	90.3	89.0 to 91.5	78.9	77.6 to 80.3	255.3	<0.001
Total	140.4	138.7 to 141.9	171.9	169.5 to 174.5	175.3	171.6 to 178.9	143.2	140.2 to 146.0	91.8	<0.001
Change										
Motor	23.9	23.2 to 24.5	24.1	22.5 to 25.7	33.8	31.2 to 36.7	11.5	10.5 to 12.5	97.7	<0.001
Cognitive	15.7	15.2 to 16.2	4.5	3.8 to 5.3	8.6	7.4 to 9.8	4.7	4.0 to 5.3	202.3	<0.001
Total	39.6	38.6 to 40.6	28.6	26.7 to 30.6	42.3	39.2 to 45.9	16.1	14.8 to 17.5	134.3	<0.001
FIM efficiency	0.44	0.42 to 0.46	0.43	0.39 to 0.47	0.54	0.49 to 0.61	0.29	0.26 to 0.33	22.0	<0.001
FIM+FAM efficiency	0.71	0.69 to 0.74	0.59	0.54 to 0.65	0.77	0.70 to 0.87	0.44	0.39 to 0.48	27.8	<0.001
*NPDS/NPCNA*										
Admission										
NPDS total score	32.2	31.7 to 32.8	24.2	23.0 to 25.4	27.7	26.0 to 29.4	26.6	25.5 to 27.7	49.0	<0.001
Care hours/week	45.4	44.9 to 46.0	39.8	38.2 to 41.3	44.6	42.2 to 4,67	43.1	41.6 to 44.5	13.9	<0.001
Care costs	£1667	£1641 to £1695	£1228	£1152 to £1302	£1452	£1336 to £1561	£1345	£1278 to £1415	46.6	<0.001
Discharge										
NPDS total score	21.3	20.7 to 21.8	14.3	13.3 to 15.3	13.4	12.0 to 14.9	21.1	19.9 to 22.2	39.7	<0.001
Care hours/week	32.4	31.8 to 33.1	24.2	22.6 to 25.8	22.7	20.4 to 24.9	35.5	33.9 to 37.0	51.1	<0.001
Care costs	£1152	£1123 to £1181	£733	£667 to £795	£684	£587 to £774	£1057	£986 to £1121	40.6	<0.001
Change										
NPDS total score	−11.0	−11.4 to −10.6	−9.9	−10.9 to −8.9	−14.3	−15.8 to −12.7	−5.5	−6.2 to −4.8	48.8	<0.001
Care hours/week	−13.0	−13.5 to −12.5	−15.6	−17.0 to −14.1	−21.9	−24.2 to −19.8	−7.6	−8.6 to −6.7	52.2	<0.001
Care costs	−£515	−£541 to −£490	−£495	−£566 to £424	−£767	−£870 to £656	−£289	−£342 to £237	25.3	<0.001
Time to offset costs of rehabilitation (months)	19.2	14.6 to 24.2	20.9	13.0 to 29.8	19.6	11.6 to 28.0	8.5	1.8 to 14.2	1.5	0.225

*Bootstrap results based on 1000 bootstrap samples.

ABI, acquired brain injury; ANOVA, analysis of variance; FIM, Functional Independence Measure; FIM+FAM, UK Functional Assessment Measure; NPDS, Northwick Park Dependency Score; NPCNA, Northwick Park Care Needs Assessment; SCI, spinal cord injury.

Between admission and discharge, change in FIM+FAM motor score was significantly different between all groups (p<0.001), except between ABI and SCI (p=1.0). Change in FIM+FAM cognitive score was significantly different between all of the groups (p<0.01) except for SCI and progressive conditions (p=1.0). Mean FIM+FAM efficiency was lowest in progressive conditions (mean 0.44) followed by the SCI group (mean 0.59) while broadly similar in the ABI and peripheral neurology groups at a mean of 0.71 and 0.77, respectively.

The differences in dependency are also summarised in table 3. In keeping with the above findings, the ABI group was the most dependent on admission. Post hoc tests showed NPDS and estimated weekly care costs to be significantly higher in ABI than all other groups (p<0.001), but there were no statistically significant differences between any of the other groups.

Between admission and discharge, reduction in dependency and care costs were significantly different between all groups (p<0.001), except between ABI and SCI (p≥0.1). The mean individually calculated time to offset the cost of rehabilitation was lowest in the progressive conditions, at 8.5 months compared with 19–20 for the other groups, but the data were widely spread with overlapping CIs and post hoc tests did not show any significant between-group differences.

### Differences between groups based on dependency at admission

The change in dependency, care needs and cost of care in the community are summarised in table 4, grouped by the level of dependency on admission.

**Table 4:** Comparison of costs and efficiency between dependency groups (n=5739)

Parameter	Low dependency (admission NPDS <10) n=699 (12%)		Medium dependency (admission NPDS 10–24) n=1607 (28%)		High dependency (admission NPDS ≥25) n=3433 (60%)		One-way ANOVA	
	Mean	95% CI	Mean	95% CI	Mean	95% CI	F	p Value
Length of stay (days)	51	47 to 54	62	59 to 64	102	99 to 104	376.3	<0.001
Cost of rehabilitation	£23 997	£22 025 to £26 089	£28 473	£27 181 to £29 731	£47 111	£45 789 to £448 314	345.0	<0.001
*NPDS/NPCNA*								
Admission								
NPDS total score	5.6	5.4 to 5.8	17.2	16.9 to 17.4	41.7	41.3 to 42.1	5401.7	<0.001
Care hours/week	15.9	15.2 to 16.6	31.9	31.3 32.4	57.1	56.6 to 57.5	4160.8	<0.001
Care costs £/week	£436	£402 to £470	£926	£897 to £954	£2109	£2083 to £2136	2466.9	<0.001
Discharge								
NPDS total score	5.1	4.6 to 5.5	9.5	9.1 to 9.9	25.7	25.2 to 26.3	913.1	<0.001
Care hours/week	11.3	10.5 to 12.0	18.7	18.0 to 19.4	39.1	38.4 to 39.8	966.1	<0.001
Care costs £/week	£306	£271 to £342	£517	−£436 to −£547	£1349	£1315 to £1384	689.9	<0.001
Change								
NPDS total score	−0.5	−1.0 to −0.0	−7.6	−8.0 to −7.2	−16.0	−16.5 to −15.5	468.0	<0.001
Care hours/week	−4.6	−5.5 to −3.8	−13.2	−13.9 to −12.5	−18.0	−18.7 to −17.3	157.4	<0.001
Care costs £/week	−£130	£−178 to −£82	−£408	£−445 to −£370	−£760	£−794 to −£726	174.2	<0.001
Efficiency								
Time to offset costs of rehabilitation (months)	27.7	15.9 to 39.7	22.3	16.9 to 29.2	14.2	9.9 to 18.8	3.7	<0.024
FIM efficiency	0.37	0.34 to 0.41	0.54	0.51 to 0.56	0.38	0.37 to 0.40	51.4	<0.001
FAM efficiency	0.70	0.64 to 0.77	0.83	0.79 to 0.88	0.58	0.56 to 0.61	54.3	<0.001

ANOVA, analysis of variance; FIM, Functional Independence Measure; FIM+FAM, UK Functional Assessment Measure; NPCNA; Northwick Park Care Needs Assessment; NPDS, Northwick Park Dependency Score.

As anticipated, LOS and the total cost of the rehabilitation episode were greatest in the high-dependency group and smallest in the low-dependency group with some twofold difference between them, and post hoc tests showed significant differences seen between all three groups (p<0.001).

The ongoing care hours and costs of care in the community remained high at discharge in the same pattern as on admission, but the reduction in care hours and costs was greater in the higher dependency groups, reflecting the higher starting levels—again with significant differences between all dependency groups (p<0.001).

Despite the higher cost of the rehabilitation, the time to offset the costs of treatment through savings in the cost of ongoing community care was shortest in the high-dependency group at 14.2 months, followed by the medium-dependency group at 22.3 months, and longest in the low-dependency group 27.7 months. But, despite the nearly twofold difference between the means for the low-dependency and high-dependency groups, the CIs were wide and the between-group ANOVA only just reached significance at p=0.024.

By contrast, FIM efficiency was highest in the medium-dependency group at 0.54 but similar between the low-dependency and high-dependency groups at 0.37 and 0.38, respectively (p=0.15). FIM+FAM efficiency was also highest in the medium-dependency group at 0.83, and again similar in the low-dependency and high-dependency groups at 0.70 and 0.58, respectively (p=0.65).

Because the data set was dominated by the ABI group, we also compared the main cost-efficiency parameters between dependency groups separately for each of the diagnostic groups—see table 5. A broadly similar pattern was seen in all the groups, with the time to offset the costs of rehabilitation being shortest in the high-dependency group (albeit with wide CIs), while FIM efficiency tended to be highest in the medium-dependency group—reaching significance in all diagnostic groups except the peripheral neurological conditions.

**Table 5:** Comparison of costs and cost-efficiency between dependency groups separated by diagnostic condition

Parameter	Low dependency (admission NPDS <10)		Medium dependency (admission NPDS 10–24)		High dependency (admission NPDS ≥25)		One-way ANOVA	
	Mean	95% CI	Mean	95% CI	Mean	95% CI	F	p Value
ABI	N=339		N=872		N=2113			
Cost of rehabilitation episode	£27 360	£24 300 to £30 305	£30 591	£28 842 to £32 292	£49 986	£48 637 to £51 406	166.3	<0.001
Reduction in weekly care costs	£152	£91 to £215	£463	£419 to £506	£760	£721 to £799	102.9	<0.001
Time to offset costs (months)	28.8	13.1 to 46.3	25.6	17.0 to 36.9	15.0	9.6 to 20.6	2.9	0.06
FIM efficiency	0.38	0.34 to 0.42	0.56	0.53 to 0.59	0.40	0.38 to 0.42	34.7	<0.001

**SCI**	**N=58**		**N=169**		**N=210**			

Cost of rehabilitation episode	£18 198	£15 179 to £21 647	£28 204	£24 812 to £31 442	£43 897	£39 825 to £48 333	30.9	<0.001
Reduction in weekly care costs	£45	£95 to £177	£407	£407 to £511	£847	£772 to £973	30.7	<0.001
Time to offset costs (months)	20.8	£9 to £58	18.7	9.7 to 27.5	22.7	10.4 to 37.2	0.10	0.91
FIM efficiency	0.37	0.28 to 0.46	0.55	0.46 to 0.63	0.36	0.31 to 0.41	8.4	<0.001

**Peripheral conditions**	**N=29**		**N=87**		**N=144**			

Cost of rehabilitation episode	£20 814	£16 539 to £26 180	£29 491	£24 338 to £35 255	£45 339	£40 021 to £51 054	11.9	<0.001
Reduction in weekly care costs	£227	£79 to £409	£405	£260 to £555	£1207	£1049 to £1372,	32.1	<0.001
Time to offset costs (months)	42.7	11.0 to 70.8	17.8	12.1 to 24.6	16.1	2.9 to 28.8	1.9	0.154
FIM efficiency	0.51	0.33 to 0.71	0.56	0.46 to 0.65	0.54	0.46 to 0.63	0.1	0.889

**Progressive conditions**	**N=72**		**N=210**		**N=344**			

Cost of rehabilitation episode	£14 118	£11 828 to £16 643	£19 476	£17 140 to £21 975	£31 991	£29 269 to £34 773	33.8	<0.001
Reduction in weekly care costs	£54	£30 to £142	£182	£94 to £266	£520	£427 to £ 616	19.3	<0.001
Time to offset costs (months)	21.6	7.4 to 36.9	13.3	6.9 to 20.3	2.8	−7.4 to 12.9	2.3	0.096
FIM efficiency	0.31	0.24 to 0.39	0.43	0.36 to 0.51	0.20	0.17 to 0.23	21.5	<0.001

ABI, acquired brain injury; ANOVA, analysis of variance; FIM, Functional Independence Measure; NPDS, Northwick Park Dependency Score; SCI, spinal cord injury.

## Discussion

Large cohort analyses of routinely collected outcome data make an important contribution to our understanding of the gains that can be made from rehabilitation in the course of real-life clinical practice, and provide the opportunity for comparing different populations and practices. This first multicentre analysis of the UK national clinical data set for specialist rehabilitation demonstrates that patients with complex neurological disability have the potential to gain from specialist rehabilitation across a wide range of conditions. It confirmed that the findings from the previous single-centre study of ABI patients^21^ were generalisable across multiple centres and a wider range of neurological conditions. Although the costs of treatment were quite high (£40 000 on average), this investment was offset by savings in the cost of ongoing care with approximately 18 months.

It should be noted that ‘specialist rehabilitation’ means something rather different in the UK from other countries. In the USA and Australia, a ‘specialist rehabilitation centre’ would be one in which the central focus of treatment is rehabilitation, often in diagnosis-specific programmes (eg, head injury, stroke or spinal cord rehabilitation). In the UK, the term ‘specialist rehabilitation’ is reserved for tertiary (levels 1 and 2) centres, serving a large catchment population (typically 1–5 million for level 1 units) and admitting a selected population of patients with highly complex rehabilitation needs, regardless of diagnosis.^16^ Thus, a stroke unit that provides rehabilitation as part of a specialist stroke programme would be classed as a level 3 (non-specialised) rehabilitation service. Patients who would progress satisfactorily within their local (level 3) rehabilitation services were not included in this analysis, which therefore represents a smaller subgroup of more complex patients, in comparison with other international rehabilitation cohorts. Our findings may nevertheless have relevance for other health systems that offer tertiary programmes of care.

The time since onset was highly skewed but, on average, very long (eg, 9 months in the ABI group) compared with other published series.^27^ Lengths of stay were also substantially longer compared with recently published series from the USA^11–13^ and Australia,^26^ so that FIM efficiency was comparatively lower (0.4 compared with 0.4–0.8 in the Australian series and 1.9–2.2 in the US series). These findings reflect the selected group of patients with complex needs admitted to the levels 1 and 2 services, many of whom had already failed to progress in their local level 3 rehabilitation services. Direct comparison of casemix-adjusted outcomes between the UK and Australian data sets^28^ confirms the preponderance of very severely disabled patients in the UK series, especially in the level 1 services. The majority of units contributing to the US and Australian data sets would be more similar to levels 2b and 3 services in the UK (Eagar K, personal communication, 2015).

Nevertheless, for a UK population with mean age 47 years in 2015, the average projected life expectancy would be approximately 40 years (males) and 42 years (females).^29^ Even if one allows an estimated 15-year reduction in respect of complex neurological disability, the mean life expectancy of this study group may be 25 years or more. Extrapolated over this period, the mean saving of nearly £500 per week (or £26 K per year) in ongoing costs of care might be expected to lead to overall life-time economic gains in excess of £650 000 or more per patient, or £3.7 billon for the whole study sample. This confirms the value of investing in appropriate specialist rehabilitation services for this group of patients. It does of course assume that the gains in independence are maintained. Evidence from a multicentre evaluation of community-based follow-up reported stability of dependency (and in some cases, further improvement) over the first year following discharge from the nine specialist levels 1 and 2a rehabilitation services in London,^30^ suggesting that this assumption is valid—and possibly even conservative—on a population basis.

Our analysis also demonstrated that cost-efficiency measured in this way was highest in the most dependent group of patients. This not only confirms the results from our previous single-centre study in patients with ABI,^21^ but also demonstrates that the reproducibility of this finding across multiple centres and different neurological conditions. FIM efficiency, meanwhile, appeared to be greatest in the medium-dependency group. This once again underlines the floor and ceiling effects the FIM in this more complex patient group and the fact that a linear trajectory of recovery cannot be assumed, nor an equal weight of items for estimating the cost of care needs.

These findings are important because, in many countries, these highly dependent patients may be denied rehabilitation if they are not expected to make significant gains on a FIM score. Thus, they emphasise the need for a range of different measures, reflecting different patient groups and their potential for change in during rehabilitation. FIM+FAM efficiency showed a similar pattern to FIM efficiency, so the additional 12 items did not necessarily improve its performance as a proxy for cost-efficiency, but they did provide a more holistic evaluation of the change in cognitive/psychosocial function, in addition to motor function, as illustrated in figure 2.

The authors recognise the following limitations to this study:

- The data were collected in the course of routine clinical practice. Despite the training provided to all units registered with UKROC, the exact level of expertise of clinicians recording the tools in each of the 62 centres is unknown. Nevertheless, the data set is reflective of real-life clinical practice, where staff experience is expected to vary.
- Because of the evolution of reporting requirements over the data collection period, the analysed sample represents less than 50% of the full rehabilitation data set. This finding was expected and comparison of demographic and baseline data suggested that the analysed sample was reasonably representative of the total population. Nevertheless, the possibility of selection bias cannot be excluded.
- The NPCNA estimates of continuing care costs are not true assessments as applied in traditional health economic studies. On the other hand, the instrument has been in use for over 15 years and is now quite widely taken up both in clinical practice and in research^24^ Experience has demonstrated it to be neither overly generous nor mean in its estimation of care needs and costs. Moreover, for the purpose of this study, we were more interested in the relative values for between-group comparison than the absolute values. Nevertheless, the estimations of cost-savings should be interpreted with some caution.
- Finally, while rehabilitation is provided through the health sector, the saving in care costs accrues to those responsible for ongoing care (typically the social care services or the patient and their family). Thus, the actual opportunity for realisation and reinvestment of the savings will depend on the local funding arrangements for health and social care.

The above limitations accepted, findings from this study add to the growing body of evidence for the cost-effectiveness of rehabilitation for patients with complex disabilities.^31^ ^32^ They confirm the potential for substantial cost-savings to be made from appropriate provision of specialist rehabilitation services for patients with complex needs, even many months after the original injury.
